# The impact of the stimulation frequency on closed-loop control with electrotactile feedback

**DOI:** 10.1186/s12984-015-0022-8

**Published:** 2015-04-09

**Authors:** Liliana P Paredes, Strahinja Dosen, Frank Rattay, Bernhard Graimann, Dario Farina

**Affiliations:** Laboratorio di Cinematica e Robotica, Fondazione Ospedale San Camillo - I.R.C.C.S., Lido di Venezia, Italy; Department of Neurorehabilitation Engineering, University Medical Center Goettingen, Goettingen, Germany; Institute for Analysis and Scientific Computing, Vienna University of Technology, Vienna, Austria; Translational Research and Knowledge Management, Otto Bock Healthcare GmbH, Duderstadt, Germany

**Keywords:** Electrotactile stimulation, Electrocutaneous stimulation, Sensory feedback in prosthetics, Stimulation frequency, Sensory substitution, Closed-loop control

## Abstract

**Background:**

Electrocutaneous stimulation can restore the missing sensory information to prosthetic users. In electrotactile feedback, the information about the prosthesis state is transmitted in the form of pulse trains. The stimulation frequency is an important parameter since it influences the data transmission rate over the feedback channel as well as the form of the elicited tactile sensations.

**Methods:**

We evaluated the influence of the stimulation frequency on the subject’s ability to utilize the feedback information during electrotactile closed-loop control. Ten healthy subjects performed a real-time compensatory tracking (standard test bench) of sinusoids and pseudorandom signals using either visual feedback (benchmark) or electrocutaneous feedback in seven conditions characterized by different combinations of the stimulation frequency (F_STIM_) and tracking error sampling rate (F_TE_). The tracking error was transmitted using two concentric electrodes placed on the forearm. The quality of tracking was assessed using the Squared Pearson Correlation Coefficient (SPCC), the Normalized Root Mean Square Tracking Error (NRMSTE) and the time delay between the reference and generated trajectories (TD_IO_).

**Results:**

The results demonstrated that F_STIM_ was more important for the control performance than F_TE_. The quality of tracking deteriorated with a decrease in the stimulation frequency, SPCC and NRMSTE (mean) were 87.5% and 9.4% in the condition 100/100 (F_TE_/F_STIM_), respectively, and deteriorated to 61.1% and 15.3% in 5/5, respectively, while the TD_IO_ increased from 359.8 ms in 100/100 to 1009 ms in 5/5. However, the performance recovered when the tracking error sampled at a low rate was delivered using a high stimulation frequency (SPCC = 83.6%, NRMSTE = 10.3%, TD_IO_ = 415.6 ms, in 5/100).

**Conclusions:**

The likely reason for the performance decrease and recovery was that the stimulation frequency critically influenced the tactile perception quality and thereby the effective rate of information transfer through the feedback channel. The outcome of this study can facilitate the selection of optimal system parameters for somatosensory feedback in upper limb prostheses. The results imply that the feedback variables (e.g., grasping force) should be transmitted at relatively high frequencies of stimulation (>25 Hz), but that they can be sampled at much lower rates (e.g., 5 Hz).

**Electronic supplementary material:**

The online version of this article (doi:10.1186/s12984-015-0022-8) contains supplementary material, which is available to authorized users.

## Background

The smooth and seemingly effortless execution of normal human grasping relies on the integration of feedforward and feedback control loops. The movement is first planned by using visual feedback and previous experience, and then executed via feedforward motor commands and online corrections through the exteroceptive and proprioceptive feedback. The operation of this sensory-motor loop is essential for the normal human motor control, learning and adaptation [1,2].

Transradial prostheses substitute morphologically and functionally the hand lost due to an amputation, thereby restoring the grasping function. For the complete and effective substitution, it is important to restore both feedforward and feedback connections between the user’s brain and the artificial device. Myoelectric control, in which the user intentions are detected by monitoring the electrical activity of his/her muscles, is a simple and reliable method to restore the feedforward pathway, and this interface has been routinely implemented for the control of prosthetic hands [3]. However, there are still no commercially available devices today providing any kind of somatosensory feedback to the user.

The missing sensory information can be restored using a method of sensory substitution. The sensory information that was captured by the receptors in the lost limb is acquired by an artificial sensor integrated into a prosthesis, and then conveyed to the brain by stimulating still intact, alternative sensory organs. The substitution can occur across sensory modalities (touch-to-sound, as in sonic feedback [4]) or within the same modality (touch-to-touch, as in electrotactile or vibrotactile feedback [5]. This approach can be used in prosthesis control. The information from the joint or force sensors embedded into the prosthesis can be transmitted to the user by stimulating the tactile sense over his/her residual limb, and thus implementing artificial proprioceptive and force feedback [6-8].

The tactile sense can be activated by using electrical stimulation. The goal of electrocutaneous or electrotactile stimulation is to activate the cutaneous afferents which lie in the epidermis and dermis skin layers. The stimulation is typically delivered via concentric electrodes since this electrode configuration generates surface currents, avoiding the unwanted activation of deeper sensory-motor structures (e.g., nerve trunks and muscles) [9]. An essential step for the effective application of electrotactile stimulation is a thorough investigation of the properties of this alternative sensory information channel. Starting in the 60’s and 70’s, several studies have been conducted to evaluate the psychometric parameters of single and multichannel electrical stimulation delivered at different places on the skin and using different stimulation waveforms, codes and electrode types [10]. For example, the sensation and pain thresholds [11-13], dynamic range [14], sensation quality [15], psychometric functions, time and spatial discrimination thresholds [14,16], recognition of discrete levels [17], and perception of continuous signals [18] have been investigated.

Contrary to visual feedback, which is normally perceived as smooth and continuous, electrotactile feedback is provided as a sequence of pulses which are delivered at a certain rate. In the context of the closed-loop control of prostheses, the rate of pulse delivery (i.e., stimulation frequency) is a very important parameter, since it directly influences the form of elicited tactile sensations (i.e., from discrete tapping to more or less fused vibrations) as well as the rate of data transfer over the feedback channel.

However, the impact of the stimulation frequency on the quality of information transfer and control via electrotactile feedback has not been investigated in the literature. In fact, there are only few studies addressing the general properties of the stimulation at different frequencies [15,17,19]. Importantly, these tests were all conducted in open loop by delivering the stimulation and asking the subject to estimate its parameters (i.e., no control action required). For example, it was reported in [17] how the frequency affected the subjective experience. Low frequency stimulation elicited a throbbing sensation, which gradually translated into vibrations (>30 Hz). The relation between pulse rate and intensity of perceived sensations was investigated in [15]. Due to a lack of systematic investigation and clinical testing, in most of the studies on the closed-loop control of prostheses [20-22], the stimulation frequency was selected based on heuristics, pilot tests and/or technical limitations.

The aim of this study was to investigate how the rate of pulse delivery influences the subject’s ability to utilize the electrotactile information as the feedback to guide the control actions. We used a closed-loop compensatory tracking task since this is a standard test bench for investigating the performance of the human operator in visually-guided [23,24] as well as electrotactile [25-28] control systems. This task allowed testing how selected feedback parameters, in this case stimulation frequency, affected the subjects’ ability to track a reference trajectory in closed-loop, which was measured by the tracking performance. The performance might be affected by different factors such as the sampling rate or the quality of perception of feedback information, where the latter refers to how well the subject could sense and interpret the delivered stimulation. The tracking with the visual feedback was used to establish an absolute reference, i.e., the benchmark performance that can be achieved with an ideal (high-fidelity and large-bandwidth) feedback interface.

## Methods

### Subjects

Ten naive, able-bodied subjects (mean (29.4) ± 1SD (4.5) yrs) participated in the experiment. The experiment was in accordance with the declaration of Helsinki and approved by the local ethics committee. Prior to the experiments, the subjects signed the informed consent.

### Experimental setup and task

The setup comprised: 1) A multichannel fully programmable electrical stimulator (RehaStim, HASOMED GmbH, Germany) connected to self-adhesive, disposable concentric electrodes (CoDe 2.0, OTBioelettronica, IT), 2) a single axis contactless joystick (HT Series, CH Products, US), 3) a standard PC with a data acquisition card (PCI-6221, National Instruments, US), and 4) a 17” monitor.

**Figure 1:**
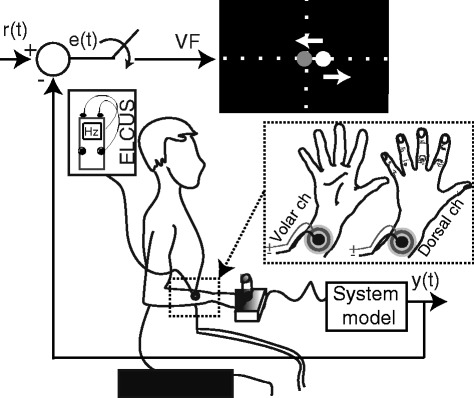
Setup for the real-time simulation of the closed-loop control. The signal r(t) is the reference trajectory, y(t) is the generated trajectory, e(t) is the tracking error, i.e., the difference between r(t) and y(t). The error was transmitted either visually (VF) or via electrotactile stimulation (ELCUS). The task for the subject was to operate the system so that the tracking error was diminished (i.e., the so-called compensatory tracking task). The electrotactile display on the forearm comprised two concentric electrodes. Minus and plus denote the cathode and anode, respectively. The sign of the error was coded by the active electrode, i.e., the dorsal channel (ch) was active for the positive and the volar channel for the negative tracking errors (spatial coding), and the pulse width was proportional to the error magnitude (intensity coding).

The experimental setup (Figure 1) implemented a closed-loop tracking task, in which the subjects operated a joystick to drive a simple dynamic system (a pure gain) along a predefined reference trajectory as accurate as possible. The tracking error, an instantaneous difference between the reference trajectory and the current system output, was delivered to the subjects via visual feedback on the computer screen or using location and intensity modulated electrocutaneous stimulation delivered through two concentric electrodes placed on the forearm. A good tracking of the reference was achieved by successfully compensating (nulling) the tracking error.

The electrocutaneous stimulator had eight stimulation channels with independently adjustable parameters (pulse width, frequency and amplitude). Two channels were used for the experiment presented here. The parameters could be set online from the PC. The stimulator generated trains of constant current, biphasic compensated pulses. The joystick was used as a command interface and provided an analog output, i.e., a voltage proportional to the joystick inclination, and this signal was digitized by a data acquisition card. The joystick was selected as a simple and consistent command interface since the goal of the present experiment was to minimize the influence of the feedforward pathway on the control performance and only focus on the properties of the feedback. For the same reasons, the centering spring was taken out from the joystick to minimize the resistance. The rubber support around the stick was enough to center the joystick when the stick was let free. Similarly, the controlled system with an ideal proportional response (no dynamics) was used in order to exclude the influence of system dynamics on the control performance and exclusively focus on the effect of the stimulation frequency on the feedback.

The PC was used to implement the real-time operation of the closed loop as explained below, and the monitor provided visual feedback, as described in the section on the Experimental protocol. An analog input was used to acquire the joystick signal, and a virtual serial port to send string commands to the stimulator via USB, modulating online the stimulation parameters. The closed-loop test bench was implemented in Matlab 2012b (MathWorks, USA) and Simulink, using Real Time Windows Target (RTWT) and Simulink 3D Animation toolboxes. RTWT signals were connected to graphical objects for providing visual feedback when needed (see Experimental protocol) [29]. A graphical user interface was implemented to setup the RTWT model parameters and control the experimental protocol. The control loop in RTWT operated at 100 Hz, and this was also the sampling rate of the reference trajectory, command input (joystick signal) and system output (generated trajectory). The tracking error was sampled and the electrotactile stimulation delivered at different rates, as specified in Experimental protocol. The refresh rate for the visual feedback was set at 50 Hz.

The error information system provided the subject with a signal indicating the direction (sign) and magnitude of the tracking error. Visual display was implemented by using two graphical markers: A grey stationary ball to denote zero tracking error (the target position) and a moving white ball whose position along the horizontal axis was proportional to the current tracking error (1 cm on the screen corresponded to 0.15 arbitrary units [au] of the signal). The ideal tracking was obtained when the moving white ball was held centered over the stationary grey ball (zero tracking error).

For the electrotactile display, the stimulation was provided through two concentric electrodes placed on the dorsal and volar side of the forearm to communicate positive and negative tracking errors (spatial coding), respectively. The electrodes were positioned on the distal-third of the forearm (Figure 1). The pulse width, and therefore the perceived magnitude of the electrotactile stimulation, was made proportional to the absolute value of the error (intensity coding). This setup was selected since it was intuitive for the subjects to map the electrode stimulation on the dorsal and volar sides of the forearm to the required joystick movements towards left and right inclination, respectively. The electrode positioning on the same side of the forearm, for example, both electrodes on the volar side, one proximal to the elbow and the other more distal, was confusing and subjects would have required a longer period of training. The electrodes were placed on the forearm since this corresponds to the envisioned application of the electrotactile feedback in hand prostheses (transradial amputation), where the electrodes will be positioned over the residual limb. Alternative placements were considered in literature (e.g., back or neck [25]), but the present configuration was selected since it leads to a self-contained system in which the feedback interface resides within the socket of the prosthesis.

The stimulation current in each electrode was kept constant at 4 milliamps (mA) and the pulse width was modulated to vary the electrocutaneous stimulation intensity. In principle, for the given electrode size, the perceived intensity of stimulation depends on the quantity of charge injected into the tissue, and can be adjusted by changing either pulse width or the current amplitude [14]. The pulse width modulation was selected in the present study since we could achieve a more gradual control of the stimulation (finer resolution) and perceived intensity with the available stimulation unit, as in [30]. The pulse amplitude was set to 4 mA based on our previous experience with electrotactile stimulation [31,32] and pilot tests in the present study. The general principle was to set the amplitude to an optimal (medium) level so that the dynamic range for the pulse width modulation stayed within the limits of the electronic stimulator (50–1000 us) across subjects and experimental conditions. If the amplitude was too high or too low, the thresholds could saturate on either side of the pulse width limits, resulting in artificially short dynamic ranges (which could not reflect the true perceptual capacities of the subjects).

The normalized tracking error (0–1 au) was mapped linearly to the stimulation dynamic range, which was defined as the range from 1.1 * ST to 0.9 * UT, where ST and UT represented the sensation threshold and the threshold for uncomfortable stimulation, respectively. During the control with electrotactile feedback, the ideal tracking was achieved when the stimulation was not delivered on either electrode (zero tracking error → no stimulation). Therefore, the instruction for the subjects was that they should steer the joystick in order to decrease and ultimately cancel the stimulation. The pulse rate was constant for the trials of an electrical stimulation condition and it was changed during the experiment according to the Experimental protocol, as explained below.

### Experimental protocol

The experiment was carried out in two sessions on different days with one day in between. The first session was the training and the second session was the evaluation. The goal of the training session was to familiarize the subjects with the experiment, allow them to understand the task, get accustomed to and understand the electrotactile feedback. The training lasted approximately 60 minutes and started with a short introduction into a compensatory tracking task (5 minutes). During the introduction, the tracking task was explained to the subjects using visual feedback and a sinusoid of 0.1 Hz as the reference. Then, the subjects tracked the same sinusoid using electrocutaneous stimulation delivered at 100 Hz and visual feedback simultaneously, to learn to interpret the former. Subsequently, the subjects practiced closed-loop control (55 minutes) with electrocutaneous feedback only by tracking pure sinusoids of 0.1 Hz and pseudorandom signals of unit-amplitude and three different bandwidths (0.1-0.2 Hz, 0.1-0.3 Hz and 0.1-0.4 Hz), five times (5 trials) per signal type. During tracking with electrocutaneous stimulation, the screen was blanked. Therefore, during the training, the subjects performed tracking tasks with an increasing level of difficulty to maintain the motivation and stimulate learning, i.e., from a simple and predictable sinusoid to faster pseudorandom signals. The training also provided an initial insight into the quality of tracking across reference trajectories of different bandwidths.

The pseudorandom signals were constructed by summing up 10 unit-amplitude sinusoids with frequencies linearly spaced within the given bandwidth and with random phases drawn uniformly from the interval 0 to 2π. The multi-sinusoids are used routinely as the reference trajectories for the closed-loop tracking [33] since they are smooth, dynamic signals with adjustable bandwidth, experienced by the subjects as randomly changing waveforms (thereby minimizing prediction across trials). Different bandwidths (maximum frequency from 0.2 Hz to 2 Hz) were tested in literature [34] for tracking using haptic interfaces. Due to a lack of information regarding electrotactile stimulation for closed-loop control, the bandwidth used in the present study was determined in pilot tests. The aim was to determine the signal dynamics that the subjects could learn to track well using the presented electrotactile display after only a short training.

On the second day, we tested the closed-loop control in different feedback conditions. The reference trajectory in the evaluation session was a pseudorandom signal of bandwidth 0.1-0.3 Hz, which corresponded to the medium level of difficulty among the tracking tasks performed during the training. The aim of the evaluation session was to test how the feedback conditions affect the control performance for the given bandwidth of the reference trajectory (level of difficulty). The feedback conditions were visual feedback (VF) and seven conditions of electrotactile stimulation. VF was delivered at the frequency of 50 Hz (graphical scene refresh rate), and it was perceived by the subjects as smooth and continuous; hence this was the benchmark condition to determine the best possible control performance (condition 1 in Table 1).

**Table 1:** **Feedback conditions in the evaluation phase**

**Condition number**	**Type of condition**
(**VF and F**_**TE**_ **/F**_**STIM**_ **)**	
1	Visual feedback (VF)
2	100/100
3	50/50
4	25/25
5	10/10
6	5/5
7	5/50
8	5/100

Notation: F_TE_ – tracking error sampling rate; F_STIM_ –stimulation frequency.

The stimulation conditions were divided in two groups. In the first group, the stimulation frequency (F_STIM_) was equal to the tracking error sampling rate (F_TE_) and both parameters were gradually reduced from 100 Hz to 50 Hz, 25 Hz, 10 Hz, and 5 Hz (conditions 2 to 6 in Table 1). With these choices, the electrotactile stimulation changed from more or less continuous (100 Hz) to a clearly discrete (5 Hz) stream of sensations, covering the most useful range for electrotactile perception [10]. In these five conditions, each stimulation pulse transmitted the instant value of the tracking error. In the second group (conditions 7 and 8 in Table 1), the error was sampled at a low frequency (F_TE_ = 5 Hz) while the stimulation frequency (F_STIM_) was increased to 50 Hz and 100 Hz. In these two cases, several pulses conveyed the same, most recently sampled value of the tracking error (sample and hold). In these two conditions, the same fidelity, low rate information about the tracking error was delivered to the subject as in the condition 5/5, but this time a higher stimulation frequency was used. This division in groups was done in order to investigate the role of the stimulation frequency and the tracking error sampling rate, independently, on the performance of the implemented closed-loop system as well as the influence of these two parameters on the interpretation of the stimulus by the subject.

**Figure 2:**
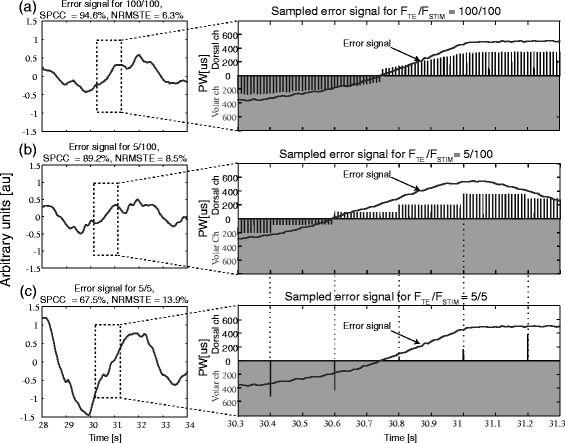
Graphical representation of the electrotactile feedback paradigm. The plots on the left are short segments from the experimentally recorded tracking errors. The plots on the right zoom into a sub-segment of the error plots and illustrate the paradigm of the electrotactile feedback. The tracking error was transmitted via the electrodes on the dorsal (upper white part of the panels) and volar side (lower gray part of the panels) of the forearm. In the conditions (F_TE_/F_STIM_) 100/100 **(a)** and 5/5 **(c)**, the error was delivered at the same rate at which it was sampled. In the condition 5/100 **(b)**, the error was sampled at 5 Hz, but delivered 20 times per sample at the frequency of 100 Hz. Note that the y-axes in the right plots represent the pulse widths (PW) of the volar and dorsal stimulation channels (ch), i.e., the height of the pulses represent the pulse width rather than the current amplitude which was constant and set to 4 mA.

The aforementioned experimental conditions are depicted graphically in Figure 2 for the three conditions (100/100, 5/5, and 5/100, F_TE/_ F_STIM_). The plots demonstrate that the sign of the error determined the active electrode (i.e., volar or dorsal channel). At the conditions with F_TE_ = F_STIM_, the intensity of each pulse was scaled according to the current value of the tracking error (Figure 2a and c). At the condition 5/100 (Figure 2b), the stimulation profiles comprised trains of pulses (N = 20) with equal pulse widths. Each pulse train transmitted the same, most recent value of the tracking error, which was sampled at the frequency of 5 Hz (compare Figure 2b vs. c).

In the evaluation session, each subject performed the tracking task five times (5 trials) in each feedback condition. Therefore, we had 50 trials per condition in total (10 subjects × 5 trials). The duration of each trial was 60 seconds. There was a 5-minute break between conditions and approximately 30 seconds of break between trials. This session lasted approximately 90 minutes in total. This protocol was selected in order to minimize the influence of subjective factors (e.g., fatigue, concentration etc.). In the pilot tests, we realized that those aspects significantly influenced the performance.

The ST and UT were determined for each subject and in each electrical stimulation condition by using the method of limits [35]. The pulse width was increased in the steps of 50 μs to find the pulse width at which the subject first perceived the stimulus (ST) and the pulse width at which the stimulation became uncomfortable (UT). ST and UT were slightly modified at any time if the subject felt discomfort or the sensorial quality diminished. The ST and UT determined during the training and evaluation sessions were in the range 70–250 μs and 500–950 μs, respectively.

### Data analysis

Offline analysis of the recorded experimental data was implemented in Matlab 2012b, and statistical tests were performed in STATISTICA 10 (StatSoft, USA).

The inputs for the data analysis were the reference and generated trajectories sampled at 100 Hz, as explained in Experimental setup and task. To evaluate the closed-loop control performance, first we estimated the TD_IO_ of the closed-loop system and afterwards evaluated the quality of tracking. The TD_IO_ was estimated by locating the time lag for which the cross-correlation function between the two signals achieved its maximum value. Afterwards, we crosschecked the validity of the estimates visually by time shifting and plotting the two signals superimposed.

The performance was also assessed by calculating the Square Pearson Correlation Coefficient (SPCC) and the Root Mean Squared Tracking Error normalized to the peak to peak value of the reference signal (NRMSTE). The expressions for SPCC and NRMSTE are presented in the Additional file 1: Appendix A. The SPCC is a measure of the overall similarity of the shapes of the two signals, and the NRMSTE indicates the average difference in their absolute values. Therefore, the two indices assessed how effective the subject was in reproducing the direction of change (increasing/decreasing) as well as the actual amplitude of the reference trajectory. The higher the SPCC and the lower the NRMSTE were, the better the subjects performed the closed-loop tracking task.

The performance results across feedback conditions and stimulation parameters (SPCC, NRMSTE, TD_IO_, UT and ST) for all subjects and trials (multivariate data) were compared using ANOVA for repeated measurements and a post-hoc multiple comparison test (Turkey’s honestly significant difference) with the p-value of 0.01 as the threshold for the statistically significant difference. The dynamic ranges, ST and UT between the volar and dorsal channel were compared using a paired t-test with p-value of 0.01.

## Results

**Figure 3:**
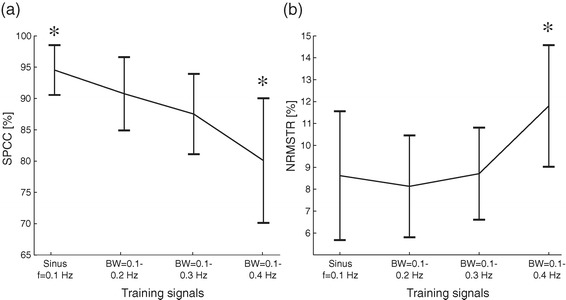
Closed-loop control performance with electrotactile feedback over all subjects during the training phase. Squared Pearson Correlation Coefficient, SPCC **(a)** and Normalized Root Mean Square Tracking Error, NRMSTE **(b)**. There was a trend of decreasing performance with the increase in the task difficulty, i.e., from simple to more complex and from slower to faster reference signals. The black asterisks above the reference signal condition of bandwidth 0.1-0.4 Hz indicate that the SPCC (NRMSTE) in this condition was statistically lower (higher) than in all other conditions.

The summary results from the training session are depicted in Figure 3. The performance gradually dropped, i.e., the SPCC decreased and NRMSTE increased, as the subjects tracked signals of increasing complexity (simple vs. pseudorandom signals) and rate of change (bandwidths). However, only the last (fastest) reference signal (bandwidth 0.1-0.4 Hz) resulted in a statistically significant decrease of SPCC and NRMSTE with respect to all the other conditions. The increase of the upper limit of the bandwidth from 0.2 Hz to 0.3 Hz did not significantly change the performance. When comparing the quality of tracking the pseudorandom trajectories (bandwidths 0.1-0.2 Hz and 0.1-0.3 Hz) against the simple sinusoid (f = 0.1 Hz), it can be seen that the SPCC significantly decreased, but the NRMSTE did not.

**Figure 4:**
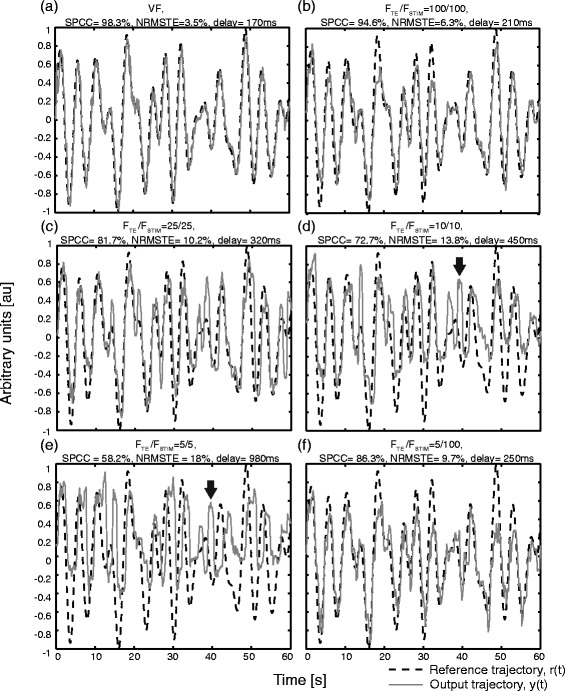
Representative tracking performance in six electrotactile feedback conditions for one subject. Reference (dotted lines) and generated trajectories (continuous lines) recorded at six conditions (F_TE_/F_STIM_) for a subject with an average tracking performance. Tracking with visual feedback was most accurate **(a)**. With electrotactile feedback **(b)**-**(f)**, the subjects could successfully follow the reference trajectory over a broad range of stimulation frequencies, although the quality of tracking decreased considerably at low stimulation frequencies. In the condition 5/5 **(e)**, the tracking was very poor and the subjects had difficulties to even identify the active electrode, i.e. error sign (see black arrows). Interestingly, the quality of tracking recovered when the low rate tracking error information was delivered at a high frequency 5/100 **(f)**.

**Figure 5:**
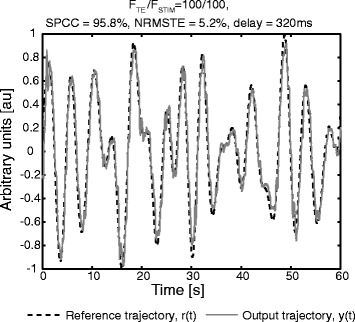
Best tracking trial with electrotactile feedback for a subject with a very good tracking performance. Reference (dotted line) and generated trajectories (continuous line) recorded for the best trial in the electrocutaneous condition 100/100 for a subject with a very good tracking performance. Tracking with electrotactile feedback was very accurate and similar to the tracking with visual feedback (compare to Figure 4a).

For the evaluation session, a representative result showing the reference and generated trajectories recorded in different feedback conditions in one subject is shown in Figure 4. With visual feedback, the closed-loop tracking was very accurate (Figure 4a). As expected, the tracking with the electrotactile feedback showed to be a more difficult task. However, for the highest stimulation frequency 100/100 (continuous sensation), the tracking was very good, and indeed for some subjects and some trials surprisingly close to the visual condition (compare Figure 4a vs. b, and see also Figure 5).

With decreasing stimulation frequency, the quality of tracking steadily deteriorated. The SPCC decreased, while NRMSTE and time delay increased. The subjects first lost the ability to correctly compensate the exact magnitude of the tracking error, while still being able to accurately estimate the direction of the change of the error. Namely, in the condition 25/25 there was still a fairly good match in the signal slopes and turning points between the reference and generated trajectories with a certain time delay between the signals, but there were large tracking errors located mainly around the peaks of the signals (Figure 4c). Finally, at the lowest stimulation frequencies (conditions 5/5 and 10/10), the subjects had difficulties in estimating the tracking error magnitude as well as the direction of change of the reference signal. This incorrect estimation was due to the wrong identification of the active electrode by subjects (sign of the error). In this case, subjects pulled the joystick in the opposite direction (Figure 4d and e. See black arrow annotations). When the low rate information about the tracking error was delivered at a high frequency (conditions 5/50 and 5/100, sample and hold), the performance improved (Figure 4f), recovering close to the level for the corresponding condition with the high stimulation frequency and high tracking error sampling rate (50/50 and 100/100, see Figure 4f vs. b). The subjects were therefore able to perform the closed-loop tracking in the conditions 5/50 and 5/100 with a similar accuracy as in the conditions 50/50 and 100/100, despite the fact that the sampling rate of the tracking error was 10 (5/50) and even 20 times (5/100) lower compared to the conditions 50/50 and 100/100, respectively.

**Figure 6:**
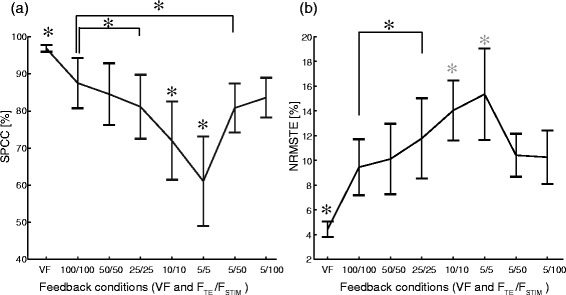
Quality of tracking averaged over all subjects and trials in each condition (VF and F_TE_/F_STIM_). Square Pearson Correlation Coefficient, SPCC **(a)** and Normalized Root Mean Square Tracking Error, NRMSTE **(b)**. In the VF condition, the performance was significantly higher than in the electrotactile feedback conditions. The closed-loop control performance first steadily worsened (decreasing SPCC, increasing NRMSTE) with the decrease of the stimulation frequency and tracking error sampling rate, but then it almost completely recovered when the low rate tracking error information was delivered using high stimulation frequencies (5/50 and 5/100). Note the abrupt performance drop in SPCC after the condition 25/25. The asterisks and horizontal bars denote statistically significant difference between the pairs of conditions. Only a black asterisk above a condition indicates that in this condition the SPCC/NRMSTE was statistically different from all other conditions. The grey asterisks in NRMSTE denote that the conditions 10/10 and 5/5 differed significantly from all other conditions, but not with respect to each other.

**Figure 7:**
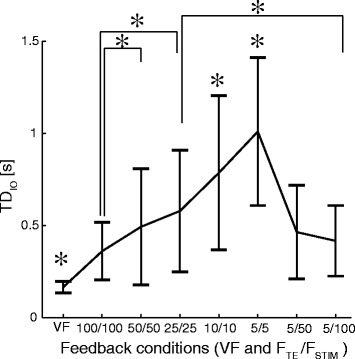
Time delays (TD_IO_) averaged over all subjects and trials in each condition (VF and F_TE_/F_STIM_). In the VF condition, the delay was significantly lower with respect to the electrotactile feedback conditions. A decrease in the stimulation frequency (tracking error sample rate) led to a sharp increase in the TD_IO_ between the reference and generated trajectory. Similarly to the other performance measures, transmitting the low rate tracking error information at higher frequencies (5/50 and 5/100) decreased the time delay (i.e., Λ shape as for NRMSTE in Figure 6b). The asterisks and horizontal bars denote statistically significant difference between pair of conditions. A black asterisk alone above the condition means that the condition was statistically different from all others.

The summary results for all subjects and all conditions are given in Figure 6 and 7. For the visual feedback, the average SPCC was 96.9% and the average NRMSTE was 4.4% (Figure 6), with a very stable performance across subjects and trials (i.e., the smallest standard deviation). Comparatively, the performance in this condition was statistically significantly higher than in all other conditions. For the electrotactile feedback, the first statistically significant decrease in SPCC (increase in NRMSTE) was registered between the conditions 100/100 and 25/25 (p < 0.0001, SPCC and NRMSTE). Thus, the frequency of stimulation could be decreased twice to the half of the previous value (i.e., from 100 to 50, and from 50 to 25) without significantly affecting the closed-loop performance with respect to the previous condition. The performance in the conditions 10/10 and 5/5 did not follow the same trend; for the SPCC, the performance in both conditions were significantly lower than the performance in all other conditions, and the same occurred for the NRMSTE except that there was no significant difference in the performance between these two conditions themselves. This change of trend is more pronounced in the SPCC plot (Figure 6a) where there was a marked decrease in the change of the SPCC at a breaking point of 25/25. Delivering the tracking error information at a low rate (as in 5/5) but at a higher stimulation frequency recovered the performance (i.e., 5/5 vs. 5/50 and 5/100). Note the characteristic V (Λ) shape of the SPCC (NRMSTE) plots (Figure 6a and b). In the conditions 5/50 and 5/100, the SPCC and NRMSTE were significantly higher and lower, respectively, than in the conditions 5/5 and 10/10 (p < 0.0001, SPCC and NRMSTE), while there was no significant difference between the performance in the condition 5/50 against the condition 50/50, or between 5/100 and 100/100. Finally, the performance in both conditions 5/50 and 5/100 were not significantly different from the performance in the condition 25/25 in SPCC and NRMSTE. A similar trend was observed for the consistency of performance across conditions. The variability increased with the decrease in the pulse rate (tracking error sampling rate), and then again decreased when the low rate tracking error information was delivered at the high stimulation frequencies. Therefore, the subjects performed better and also more consistently when the feedback was delivered at high frequencies of stimulation (>25 Hz).

Large delays in the closed-loop tracking appeared for the electrotactile feedback against the visual feedback and for decreasing electrocutaneous stimulation frequency (Figure 7). The average time delay (mean ± 1SD) was 164 ± 37.9 ms for the visual feedback and almost twice higher for the electrotactile condition with 100/100 (359.8 ± 162.4 ms), and the delay increased to as much as 1008 ± 407.7 ms in the condition 5/5. When delivering the low rate tracking error information using a high stimulation frequency, the delay markedly decreased. There were no statistically significant differences between the delays in conditions 5/50 and 5/100 and the corresponding conditions with the high stimulation frequency and high tracking error sample rate (50/50 and 100/100). A similar trend was observed for the consistency of time delays across conditions. The variability increased with the decrease in stimulation frequency (tracking error sampling rate), and then again decreased when the tracking error was sampled at the same low rate, but delivered using high stimulation frequencies.

As it can be inferred from the plots of SPCC, NRMSTE and TD_IO_ (Figure 6 and 7), the performance measures were correlated. There were a significant positive correlation between the NRMSTE and time delay (r^2^ = 65% at p < 0.01), and a significant negative correlation between SPCC and NRMSTE (r^2^ = −81.5% at p < 0.01), and between SPCC and time delay (r^2^ = −77.1% at p < 0.01).

The average sensation threshold and the uncomfortable threshold for stimulation (mean ± 1SD) over all subjects and electrotactile conditions for the electrode placed on the volar side (ST = 125.8 ± 50.2 μs; UT = 826.6 ± 192.8 μs) were lower than for the dorsal electrode (ST = 151 ± 57.2 μs; UT = 839.2 ± 175.9 μs), and this difference was statistically significant (p < 0.00001). The dynamic range defined as UT – ST was significantly higher (p < 0.001) at the volar side (700 ± 186 μs) than at the dorsal side (688.2 ± 164.6 μs).

Across the electrotactile stimulation conditions, the average ST and UT at both electrodes tended to increase for lower stimulation frequencies. For example, for the volar electrode the ST and UT at the condition 100/100 were significantly lower than in all other conditions, except in 5/100 for ST and 50/50, 5/50 and 5/100 for UT; in 50/50 the ST and UT were significantly lower only with respect to the condition 5/5. In contrast, the dynamic range was similar between the conditions for both electrodes, i.e., no statistically significant differences between the dorsal and the volar channel, only the dynamic range in condition 5/5 significantly differed from the condition 100/100 at the volar channel. For the detailed information on the statistical differences, see Table 2 for ST, Table 3 for UT and Table 4 for dynamic ranges of the volar and dorsal electrodes.

**Table 2:** **Sensation thresholds (ST) for the volar and dorsal channels**

**Electrotactile condition**	**100/100**	**50/50**	**25/25**	**10/10**	**5/5**	**5/50**	**5/100**
100/100							
50/50	*						
25/25	*/°						
10/10	*/°						
5/5	*/°	*/°					
5/50	*		/°	*/°	*/°		
5/100		*	*/°	*/°	*/°		
Dorsal (°)	130 ± 51	149 ± 47	164 ± 73	167 ± 59	177 ± 68	138 ± 35	133 ± 43
Volar (*)	109 ± 51	128 ± 48	132 ± 49	137 ± 50	140 ± 54	121 ± 46	114 ± 47

Sensation thresholds (Mean ± 1SD) for the volar and dorsal channels in each feedback condition and statistically significant differences among the sensation thresholds in the different conditions for the volar (*) and dorsal (°) channels.

**Table 3:** **Uncomfortable thresholds (UT) for the volar and dorsal channels**

**Tactile condition**	**100/100**	**50/50**	**25/25**	**10/10**	**5/5**	**5/50**	**5/100**
100/100							
50/50							
25/25	*/°						
10/10	*/°						
5/5	*/°	*					
5/50							
5/100				/°	/°		
Dorsal	812 ± 202	839 ± 188	848 ± 175	855 ± 154	865 ± 124	840 ± 183	815 ± 197
Volar	785 ± 247	812 ± 206	835 ± 184	848 ± 175	860 ± 139	820 ± 192	820 ± 192

Uncomfortable thresholds (Mean ± 1SD) for the volar and dorsal channels in each feedback condition and statistically significant differences among the uncomfortable thresholds in the different conditions for the volar (*) and dorsal (°) channels.

**Table 4:** **Dynamic ranges for the volar and dorsal channels**

**Electrotactile condition**	**100/100**	**50/50**	**25/25**	**10/10**	**5/5**	**5/50**	**5/100**
100/100							
50/50							
25/25							
10/10							
5/5	*						
5/50							
5/100							
Dorsal	682 ± 186	691 ± 177	684 ± 166	688 ± 146	688 ± 121	702 ± 173	682 ± 181
Volar	676 ± 232	684 ± 203	703 ± 180	711 ± 178	720 ± 137	699 ± 182	706 ± 185

Dynamic ranges (Mean ± 1SD) for the volar and dorsal channels in each feedback condition and statistically significant differences among the dynamic ranges in the different conditions for the volar (*) channel.

## Discussion

We have investigated the influence of the stimulation frequency and tracking error sampling rate on the closed-loop control performance. These parameters are essential for the tuning of closed-loop prosthetic systems relying on electrotactile feedback. The results of the present study demonstrate that the stimulation frequency is more important for the control performance than the tracking error sampling rate, within the ranges tested (5–100 Hz). The likely reason for this is that the stimulation frequency critically affects the quality of perception of the electrotactile stimulation. The closed-loop tracking task revealed that the change in perception with frequency was such that it substantially impacted the subjects’ ability for closed-loop control. In the following, we discuss the reasons pointing out to the connection between the performance, stimulation frequency and perception, speculate on the possible mechanisms responsible for the changes in perception, and suggest implications for the practical application of the study outcomes.

Decreasing the frequency of stimulation (from 100 Hz to 5 Hz) also reduced the rate of sampling of the tracking error (conditions 2 to 6). Therefore, the feedback channel gradually transformed from an almost continuous to a clearly discrete stream of information, providing therefore less and less information about the tracking error to the subject. However, it is unlikely that this decrease in the tracking error sampling rate was responsible for the concurrent drop in performance. The SPCC dropped abruptly in the conditions 5/5 and 10/10, although the error sampling rate at these frequencies was more than 15 and 30 times faster, respectively, than the upper limit of the bandwidth of the reference trajectory (0.3 Hz). And indeed, when the same fidelity, low-rate information about the tracking error was delivered using a higher stimulation frequency (conditions 5/50 and 5/100), the performance recovered and was similar to the performance in the conditions in which the tracking error was sampled and delivered at the high rates (50/50 and 100/100). Therefore, we assume that the main reason for the decrease in performance was not the transition of communication/control loops from a continuous to a (low rate) discrete state, but the fact that the stimulation frequency critically affected the quality and localization of the elicited tactile sensations. The higher stimulation frequencies resulted in sensations that were easier to discriminate by the subjects (finer perceptual resolution). At low stimulation frequencies of 5 and 10 Hz, the performance dropped significantly because the subjects had difficulties in correctly perceiving the magnitude and sign (active electrode) of the error.

The previous conclusion was also supported by the verbal feedback received from the subjects. They reported that the high frequency of stimulation at 100 Hz felt as a superficial and well-localized tingling and/or pressure-like sensation. Afterwards when decreasing the stimulation frequency to 50 Hz and 25 Hz, they reported vibrating sensations which were still sensed superficially. At low frequencies of 10 Hz to 5 Hz, the subjects experienced tapping sensations which were less clear and felt deeper, and they reported that sometimes even the most fundamental information about the tracking error, i.e., the currently active channel (error sign), was difficult to identify. In addition, during the static tests (threshold determination) the subjects reported that low frequency stimulation was felt as natural tapping sensation, while during the dynamic tracking task they experienced the lower frequencies as less comfortable. This was due to the poorer performance resulting in larger tracking errors. The stimulation was therefore delivered with the pulse widths in the higher segments of the dynamic range, closer to the upper threshold, eliciting thus stronger sensations closer to the level of discomfort.

The delays, TD_IO,_ obtained for the tracking with visual feedback and for the electrotactile feedback at high frequencies (100 and 50 Hz) were close to those reported in the literature [8]. However, the decrease in stimulation frequency produced a substantial increase in the TD_IO_. As an input–output measure, this time delay characterizes the overall behaviour of the closed-loop system, indicating that it became slower in responding to the reference input. However, it does not provide insight into the behaviour of the individual components within the loop (e.g., eventual time delays in the human operator response). This delay increase cannot be explained by the transition from a more continuous (100/100) to a discrete control (5/5). During this process the sampling time was increased from 0.01 to 0.2 s and this increase was too small to explain the delays of up to approximately 1 s in the condition 5/5 [36]. Instead, we assume that this was likely the consequence of a poor perception of electrotactile stimulation at the low stimulation frequencies, compromising the subjects’ ability to interpret the feedback and react timely with an appropriate control action. Due to a poor perception, the time needed for the cognitive processing of the feedback information might have increased or the subject changed the response strategy (e.g., acting more conservatively) due to higher uncertainty. A different analysis is necessary to obtain insights into these mechanisms, i.e., the methods for the identification of the systems in closed-loop [37,38], which is an important step, but outside the scope of the current study.

The changes in the perception of the electrotactile stimulation might be due to the fact that the afferent channel could have been activated differently at different stimulation frequencies. The electrophysiological and neural mechanisms of activation and information processing in the tactile system during electrotactile stimulation are still largely unexplored [39]. Better perception and/or finer resolution at higher frequencies could be due to a temporal summation of the individual (electrically produced) stimuli in the neural networks that process the tactile inputs, i.e., higher pulse rates produced higher rates of firing of the activated cutaneous afferents [39]. However, it could also be that different stimulation frequencies activated different fiber types. Some evidence for this possibility can be found in the literature about transcutaneous neuromodulation (TENS) methods [40]. The typical stimulation frequencies used in TENS (2–150 Hz) correspond closely to the range used in this study (5–100 Hz). It was suggested in TENS literature that the large-diameter Aβ and small-diameter Aδ nerve fibers respond preferentially to a high (90–130 Hz) and low frequencies (2–5 Hz) of stimulation, respectively. This could also explain the very different quality of elicited sensations reported by the subjects at the electrotactile feedback conditions 5/5 and 10/10 with respect to the 100/100 condition. This issue is still highly controversial and a definite answer is unknown [41,42]. Finally, it could be that certain stimulation frequencies generated firing patterns that were similar to the ones which are produced naturally in specific fiber types. For example, Pacinian corpuscles respond to vibrations in the range of 40–800 Hz. When the fibers innervating these organs are activated at non-natural frequencies (e.g., 5 Hz), this could cause confusion in neural perception or the information could be simply blanked out by the neural processing [43]. Some of these factors or a combination of them could explain the changes in perception at different stimulation frequencies, leading to a lower quality of elicited sensations at the conditions with low stimulation frequencies. Instead, when the data were sent at the higher frequencies, the subjects could perceive the magnitude and direction of the error faster and better, and thus respond more promptly with a corrective action. Hence, the performance increased and the delay decreased at high stimulation frequencies.

The results have demonstrated that the electrotactile feedback could be successfully utilized by the subjects to achieve a good performance during a closed-loop control task. This was possible after a relatively short training (duration of 60 minutes) and over a wide range of stimulation frequencies. Although, as explained before, the stimulation frequency critically affected the information transfer over the feedback channel, it seems that the human cognitive control of the sensory motor loop is rather robust. The frequency could be reduced significantly (from 100 Hz to 25 Hz) with a fairly gradual drop in average performance, e.g. from 87.5% to 81.1% in SPCC. The most prominent decrease was registered only when the frequency was set to as low as 10 Hz and 5 Hz, e.g. 71.9% and 61.1% in SPCC (Figure 6a). Even at those low rates, the subjects could perceive and compensate the errors to a certain extent, so that the generated trajectory roughly followed the shape of the desired (reference) signal.

The main aim of this study was to investigate the electrotactile feedback conditions, and the tracking with the visual feedback was tested in order to establish the benchmark performance. The latter facilitates the qualitative appraisal of the electrotactile tracking results. In that sense, the present study demonstrates that a dynamic control task can be successfully accomplished using electrotactile feedback (basic feasibility) and also that with the high stimulation frequency (e.g., 100/100), the quality of tracking can be close to the benchmark levels achieved with visual feedback (Figure 4a and b and Figure 5). This is a promising outcome, suggesting that the electrotactile feedback can result in good performance during a dynamic task. Therefore, the electrotactile stimulation can be applied to close the loop in the control of hand prosthesis, and it seems that it might be able to provide assistance to the user even when the visual feedback is completely absent (e.g., the user does not look at the hand). Our focus was to investigate the impact of feedback parameters on the closed-loop control performance, since this is the context of interest for the control of prostheses equipped with electrotactile feedback. Therefore, the perception of electrotactile stimulation was directly evaluated using only the basic static tests, i.e., determining the thresholds and dynamic ranges to implement the electrotactile intensity coding. However, the control performance across feedback conditions provided important indirect insights into the quality of perception, as explained above.

The static perceptual tests also provided a further clue about the possible nature of the changes in perception across stimulation frequencies. Specifically, the fact that the determined dynamic ranges for both electrodes were not significantly different across the conditions implies that the performance drop was not simply due to a lower range of the stimulation intensities available for coding of information (tracking error) on one of the electrodes. The psychometric parameters still differed between the volar and dorsal electrode, indicating a somewhat higher sensitivity of the volar side (lower ST, UT and higher dynamic range). The volar side could be therefore preferred for the placement of the electrodes in the actual application of the electrotactile feedback in prosthetics. However, the absolute differences between both channels were rather small (~25 μs for ST, 13 μs for UT and 11 μs for dynamic range) and further tests are necessary to confirm this initial recommendation (e.g., using prosthesis and/or more sophisticated psychometric methods [44]).

It seems that the human operator can perceive and tolerate a similar range of stimulation intensities across a wide range of electrotactile stimulation rates in both sides of the arm. However, as explained above, this does not mean that the subjects were able to discriminate different magnitudes of stimulation with the same precision in all conditions. In other words, the difference in performance could be explained by a change in the size of just noticeable differences (JNDs). JND is defined as the smallest change in the stimulation parameter with respect to a certain baseline value that can be felt by the subject. The subjects indicated that at high frequencies they were able to better perceive the changes in the electrotactile stimulation intensity and thereby reacted faster to properly compensate for the error. This implies that JNDs might be smaller at higher stimulation frequencies and the subjects were therefore able to discriminate more levels within the dynamic range, resulting in finer perceptual resolution and better control performance. This was also supported by the determined sensation thresholds (ST), which were somewhat lower at higher frequencies, suggesting a better sensitivity in these conditions. However, this is only a hypothesis based on the verbal reports and indirect cues (ST and UT) and it needs to be tested experimentally by determining a sequence of JND levels for different stimulation frequencies.

The increase of the ST and UT at the lower frequencies is a known phenomenon, likely reflecting the effect of temporal summation/integration of the individual pulses [45]. This increase was rather small (the maximum mean increase for ST was 31.8 μs for condition 6 (5/5) with respect to condition 2 (100/100) for the dorsal channel and for UT was 75.4 μs between the same conditions for the volar channel) and statistically significant only in a limited number of cases (Tables 2 and 3). Importantly, in all of the subjects and conditions, the UT was less than the technical limit of the stimulation unit. Finally, the perceptual dynamic ranges (Table 4) were consistent across conditions, spanning a large segment (on average 75.7%) of the full dynamic range provided by the stimulation unit (50–1000 us). All these points confirm that the current amplitude of 4 mA was indeed a good choice, since all subjects operated across full individual perceptual ranges in all conditions. We therefore believe that the aforementioned psychometric parameters had a minor (if any) influence on the trend in the control performance across conditions. Rather, the trend reflects a more general mechanism for the modulation of the perceptual experience with stimulation frequency and might be related to the effective perceptual resolution of the feedback channel (as explained in the previous paragraphs).

The subjects accepted electrocutaneous stimulation without difficulty, except for an initial apprehension in some subjects which was easily overcome during the training phase. Contrary to visual feedback which could be utilized immediately with high performance, the training was essential in the case of the electrotactile feedback. This was true even for the simplest case of the sinusoidal signal, which is a periodic and easily predictable waveform. The short training of 60 minutes with gradually increasing task difficulty was enough for the subjects to reach a stable tracking performance of the pseudorandom trajectories with frequency components up to 0.3 Hz. This was in fact the limit of what could have been accomplished during the training session, since the performance significantly deteriorated if the bandwidth was only slightly increased (up to 0.4 Hz). Note that this bandwidth is within the activities of daily living of a prospective user of a prosthesis (e.g., few seconds to open and close the prosthesis), but also less than the assumed bandwidth of the human manual control during compensatory tracking of multi-sinusoids as well as other trajectories (e.g., filtered noise) using visual feedback [46,47]. The frequency domain analysis of the human operator showed that the bandwidth, as determined from the frequency-gain characteristics, is influenced by multiple factors (e.g., subjects training, controlled system dynamics, reference trajectory etc.). Nevertheless, previous studies have demonstrated that the closed-loop system bandwidth of the skilled subjects during visual tracking could reach between 1 and 2 Hz [48]. Regarding the electrotactile feedback, further training would likely allow the tracking of faster reference signals with good performance, but the ultimate limit of the electrotactile closed-loop still needs to be determined. The latter demands a dedicated analysis [47] and was outside the scope of the present study.

It is likely that using a reference trajectory with a higher bandwidth (e.g. 0.1-0.4Hz) during the evaluation session would not have changed the main conclusions. The task would have become more difficult, leading to a decrease in performance in all conditions, but the trend would remain similar across the conditions since it seems to reflect an inherent feature/mechanism of electrotactile stimulation (i.e., change of perception with stimulation frequency). This also holds for the other parameters of the experimental setup that had to be selected among many possible options, such as, the electrode type (concentric vs. unifocal) and size, placement, modulation method (pulse width vs. amplitude), but also subject characteristics (able-bodied vs. amputees, age group etc.).

There are studies indicating that the tactile perception in some amputees is not impaired [49], [50], also after the targeted muscle and sensory reinnervation [51]. Still this is likely patient specific and strongly depends on the amount and style of the prosthesis use. This can affect the condition of the stump skin due to prolonged socket application and/or the perception and processing of the tactile sensations (e.g., tuning to register subtle tactile feedback cues). Also, it should be taken into account that other more complex perceptual abnormalities [52] might play an important role when testing closed-loop control with electrotactile feedback in amputees. For example, some subjects might suffer from chronic pain which is known to affect both the perception and action. In that sense, the effect of the phantom limb pain onto the application of electrical feedback (e.g., hypersensitivity) and overall control performance might be more pronounced than the modulation of perception due to frequency of stimulation. Similarly, we assume that the test in other age groups (e.g., older subjects) would reveal the same trend since the mechanism responsible for the change in control performance is rather general. However, more training might be necessary to achieve the same baseline results, due to possibly less dexterous sensory-motor control.

The fact that the stimulation frequency had a strong impact on the TD_IO_ of the closed-loop system is relevant information. One approach to better understand the behaviour of the human operator would be to model his/her response using system identification methods developed specifically for closed-loop configurations [38]. The change in the dynamics of the human controller (e.g., response time constants, pure time delays) could affect the overall system behaviour, even its stability, especially when considering that other inert systems will be integrated within the feedback loop (e.g., prosthesis). These important aspects will be addressed in a dedicated study.

## Conclusions

This study characterizes the behaviour of a human controller with respect to the stimulation frequency and the tracking error sampling rate over the electrotactile feedback channel, which is directly relevant for the selection of optimal system parameters for somatosensory feedback in upper limb prostheses. Specifically, the results suggest that the stimulation frequency is a more important parameter for the control performance, critically affecting the quality of perception. Practically, this means that the feedback variables of interest (e.g., grasping force, hand aperture) should be delivered at a high frequency of stimulation (e.g., 100 Hz, at least > 25 Hz) in order to assure the best perception of the electrotactile information, leading in turn to the best control performance and smallest response delays. At the same time, the feedback variables could be sampled at a significantly low rate (e.g., 5 Hz) without affecting the performance and response delays. This low rate of information transfer could simplify the design and processing within the embedded hand prosthetic controller (e.g., acquisition, signal conditioning and amount of information to transmit). The potential drawbacks of using high frequency stimulation, such as stronger interference with myocontrol [31] and faster habituation [53], could be overcome using known techniques (intermittent stimulation [53], artifact suppression [31]). This study therefore contributes to the further understanding of the basic properties and mechanisms of closed-loop control using electrotactile feedback and also provides practical guidelines on how to deliver the feedback more effectively to the prosthesis user.

## Additional file

Additional file 1: Appendix A. Expressions for Squared Pearson Correlation Coefficient (SPCC) and Normalized Root Mean Squared Tracking Error (NRMSTE).
