# Supplementary material for: The impact of the stimulation frequency on closed-loop control with electrotactile feedback
Source: J Neuroeng Rehabil. 2015 Apr 9;12:35. doi: 10.1186/s12984-015-0022-8 (PMC4403675; doi:10.1186/s12984-015-0022-8)
Supplement: Additional file 1: Appendix A. — Expressions for Squared Pearson Correlation Coefficient (SPCC) and Normalized Root Mean Squared Tracking Error (NRMSTE). [file 12984_2015_22_MOESM1_ESM.pdf]

# Additional files

### Additional file 1 – Appendix A

The expression Squared Pearson Correlation Coefficient (SPCC) is:

$$SPCC(\%) = CORR^2 * 100$$

with $CORR = \frac{\mathrm{cov}(r(t), y(t))}{\sqrt{\mathrm{cov}(r(t), r(t)) * \mathrm{cov}(y(t), y(t))}}$,

The expression Normalized Root Mean Squared Tracking Error (NRMSTE) is:

$$NRMSTE(\%) = \frac{\sqrt{\sum_1^N (r(t) - y(t))^2}}{\max(r(t)) - \min(r(t))} * 100,$$

where $N$ is the number of samples, *cov* denotes covariance, and *r(t)* and *y(t)* are the reference and generated trajectory, respectively.
